# PitNETs and the gut microbiota: potential connections, future directions

**DOI:** 10.3389/fendo.2023.1255911

**Published:** 2023-11-06

**Authors:** Ding Nie, Chuzhong Li, Yazhuo Zhang

**Affiliations:** Beijing Neurosurgical Institute, Capital Medical University, Beijing, China

**Keywords:** intestinal flora, gut microbiota, pituitary neuroendocrine tumor (PitNET), gut-brain axis, pituitary tumor

## Abstract

The role of the gut microbiome has been widely discussed in numerous works of literature. The biggest concern is the association of the gut microbiome with the central nervous system through the microbiome-brain-gut axis in the past ten years. As more and more research has been done on the relationship between the disease of the central nervous system and gut microbes. This fact is being revealed that gut microbes seem to play an important role from the onset and progression of the disease to clinical symptoms, and new treatments. As a special tumor of the central nervous system, pituitary neuroendocrine tumors (PitNETs)are closely related to metabolism, endocrinology, and immunity. These factors are the vectors through which intestinal microbes interact with the central nervous system. However, little is known about the effects of gut microbes on the PitNET. In this review, the relationship of gut microbiota in PitNETs is introduced, the potential effects of the gut-brain axis in this relationship are analyzed, and future research directions are presented.

## 1 Introduction

A variety of tumors can occur within sella, including meningiomas, germ cell tumors, and metastases, but primary tumors of the adenohypophysial gland are the most common (1). In 2022, the World Health Organization (WHO) adopted PitNET according to tumor cell lineage, cell type, and related characteristics to describe this type of tumor formerly known as a pituitary adenoma (PA) (2). The process of formation of such tumors is generally thought to be caused by the clonal amplification of a single abnormal cell caused by a somatic genetic mutation or chromosomal abnormality, but the exact mechanism remains unclear (3). These tumors are also known as nonfunction pituitary neuroendocrine tumors (NF-PitNETs), prolactinoma, somatotropinoma, corticotropinoma, thyrotropinoma, and gonadotropinoma, depending on whether they cause hormone overproduction (4). Although these tumors are usually non-malignant, their high prevalence, which occurs in 10% of the population, remains a threat to human health (5). Even with the rapid development of neuroendoscopic surgery, the effective treatment of certain subtypes (invasive/functional) of PitNET has been a difficult problem for clinicians (6). In the further study of the pathogenesis and development of PitNETs and the emerging treatment methods including chemotherapy drugs and immunotherapy, the study of intestinal microbiota in patients with PitNETs will naturally become an interesting direction, and indeed some researchers including us have done so (7–10). As the subject of extensive research in the last decade, the gut microbiota is associated with and plays a role in the occurrence or development of numerous diseases (11–13). With further research, the concept of the “gut-brain axis” has been proposed, which is a bidirectional communication system with complex signaling mechanisms, including the vagus nerve, enteric nervous system, immune system, and release of microbial metabolites (14, 15). These studies have shown that the gut microbiome regulates neurobehavioral characteristics and endocrine function and can communicate with the central nervous system. Given the role of PitNETs, a kind of disease of the central nervous system, in the endocrine system, the potential association with gut microbiota is of interest (10). However, to the best of our knowledge, the relationship between gut microbiota and PitNETs, or even the pituitary gland, has not been clearly defined, although the hypothalamic-pituitary-adrenal (HPA) axis has been studied as a hot spot pathway in the brain-gut axis for many years (16, 17). Encouragingly, a large body of research seems to hint at a relationship between PitNETs and gut microbes. In solid tumors such as lung cancer and melanoma, changing the composition of a patient’s gut microbiota based on fecal microbial transplantation or antibiotic administration can enhance the efficacy of immune checkpoint blocking (ICBs) (18). A study showed the antitumor effects of ICB therapy in Cushing’s disease mice (19). The role of gut microbes in the treatment of central nervous system diseases such as depression has been demonstrated (20). These studies have led to the belief that gut microbes may be new therapeutic targets for PitNETs. This review reviews the research progress of the relationship between gut microbiota and PitNETs and summarizes the possible mechanism of gut microbiota involvement in PitNETs.

## 2 Intestinal flora in patients with PitNETs

We reviewed all the English literature up to October 2023 and found a total of 5 studies exploring the relationship between gut microbiota and PitNETs (7–10, 21). Of these five studies, four of the main studies were in patients with somatotropinoma, while Hu, Et al. described the microbiota of patients with invasive/non-invasive tumors and revealed the difference in microbiome composition between the two groups (**Table 1**). In these studies, the authors all described the intestinal microbiota composition of patients with PitNETs, revealing that the diversity of intestinal microbiota in PitNETs patients is different from that in the normal population. The richness and diversity of intestinal microbiota species can reflect the health or disease status of the human body and can even be used as predictors of disease prediction (22, 23). Lin et al. used a resume random forest classifier model to distinguish patients with Growth hormone (GH)-secreting pituitary tumor from healthy controls based on gut microbiota composition (9). Serdar et al. successfully distinguished individuals with abnormally high levels of insulin-like growth factor-1 (IGF-1) and accurately identified patients with acromegaly by using a machine learning model using microbiome composition (7). Hu, J. et al. established a classification model and identified 10 species that could be used to predict the occurrence of PitNETs, including *Oscillibacter* sp. *57_20*, *Fusobacterium mortiferum*, and *Clostridium innocuum (* 10). With the development of metagenomic sequencing and the expansion of sample sizes, for example, as the gut microbiome is an early predictor of disease in other types of diseases, this same effect may be applied to patients with PitNETs (24, 25). This allows for earlier diagnosis and treatment. Regrettably, only one of these studies, involving animal experiments, made a preliminary exploration of the relationship between gut microbiota and PitNETs but was limited to the observation of phenomena that didn’t reveal a possible pathway (8). At the same time, considering all literature reports, the description of intestinal flora composition in patients with different types of PitNETs remains to be explored by researchers. It is worth mentioning that Gavin et al. studied sphenoidal sinus flora in pituitary apoplexy patients and observed differences in the diversity of sphenoidal sinus flora between pituitary apoplexy patients and NF-PitNETs patients (26). Ye et al. recently sequenced 16SrRNA from PitNET tissues and speculated that the pathogenesis and development of tumors may be related to the behavior of bacteria in tumors (27). These studies mean that the microbes associated with PitNETs may not be limited to the gut, and future prospective studies are warranted.

**Table 1:** Intestinal flora associated with PitNETs.

Author	Sample type	Study population	Sample size (n)	Sequencing methods	Key findings	Limitation	Country	Year	Reference
Hu et al.	Fecal	HC: IPA: NIPA	15:13:16	Metagenomic	1. significant alterations in the microbial community among PA patients. 2. The enrichment of *Clostridium inoculum*, along with the reduced abundance of *Oscillibacter sp. 57_20* and *Fusobacterium mortiferum*, were observed both in the IPA and NIPA groups compared to the control group.	No animal level studies, no mechanism studies	China	2022	(10)
Nie et al.	Fecal	Healthy controls :GHPA: NFPA	25:25:25	16SrRNA	1. The β-diversity of intestinal microbiota was different among the three groups. 2. At the genus level, *Bacteroides, Biautia, Enterococcus, Megamonas*, as well as other genera were differentially represented among the three groups.	No mechanism studies	China	2022	(8)
Lin et al.	Fecal	GHPA: Healthy controls	28:67	16SrRNA Metagenomic (9:10)	1. Patients with GHPA had reduced microbiota diversity and increased levels of *Oscillibacter* and *Enterobacter genera*. 2. Strong association between *Enterobacter* and GH/IGF-1 axis in disease.	No animal level studies, no mechanism studies	China	2022	(9)
Serdar et al.	Oral and fecal	Acromegaly : Healthy controls	15:15	16SrRNA	1. There was good agreement between fecal and oral microbiota in patients with acromegaly. 2. Oral microbiota diversity was significantly increased in patients with acromegaly. 3. In the fecal microbiota, the *Firmicutes/Bacteroidetes* ratio was lower in patients with acromegaly than in healthy controls	No animal level studies, no mechanism studies	Turkey	2022	(7)
Aysa et al.	Fecal	Acromegaly : Healthy controls	7:10	16SrRNA	1. A significantly lower bacterial diversity in the patients with acromegaly. 2. *Bacteroidetes* phylum was pre-dominating in the patient group, and *Firmicutes/Bacteroidetes* ratio was altered significantly.	No animal level studies, no mechanism studies	Turkey	2021	(21)

HC, healthy subjects; IPA, invasive pituitary adenoma; NIPA, noninvasive pituitary adenoma; GHPA, growth hormone-secreting pituitary; NFPA, growth hormone-secreting pituitary adenoma; PA, pituitary adenoma; GH, growth hormone; IGF-1, insulin-like growth factor-1.

## 3 Gut microbiota and the gut-brain axis

**Figure 1:**
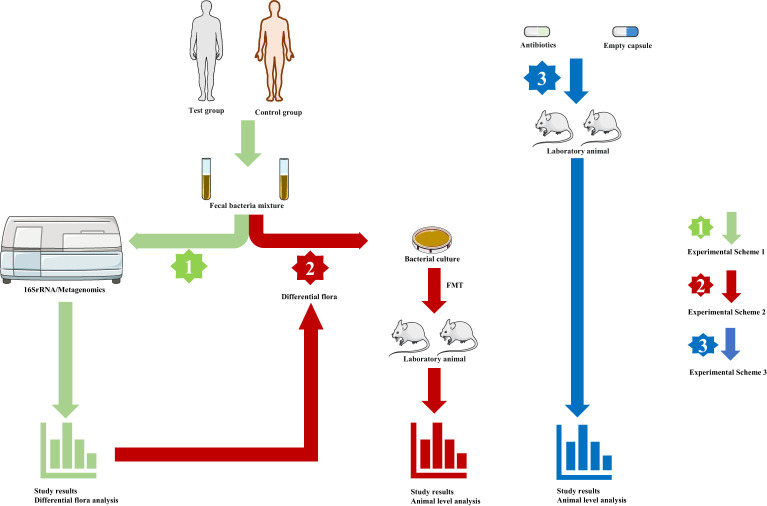
The main models of gut microbiota research. (Green arrow, experimental scheme 1, human fecal samples were collected and sequenced to analyze the characteristics of intestinal microbial composition and metabolism analysis. Red arrow, experimental scheme 2, based on experiment scheme 1, fecal bacteria were transplanted into experimental animals, and the corresponding index changes were observed at the animal level fecal. Blue arrow, experimental scheme 3, Depletion of gut microbes through antibiotics, and studies were conducted in normal and germ-free animals antibiotics.).

The known gut microbiota in healthy people is not less than 1000 species, with more than 3 million genes (28). Similar to a fingerprint, each individual has its unique gut microbiota as determined by host genotype, initial colonization by vertical transmission at birth, and dietary habits (29–31). At the same time, age, living environment, and the use of antibiotics can affect the structure of intestinal microbiota (22). For decades, it has been studied scientifically as a key factor in the maintenance of human health and the mechanism of disease occurrence and development and has been confirmed in numerous studies (32, 33). So far, studies on gut microbiota and disease have been divided into several mainstream models (**Figure 1**). First, researchers sought to characterize changes in the composition and distribution of the gut microbiota in disease states. For example, Birol et al. described differences in gut microbiota composition between patients with idiopathic focal epilepsy and healthy volunteers (34). As sequencing methods have updated (from 16SrRNA to metagenomics), researchers have moved from a genus level of the gut microbiota to a more precise species level providing a basis for further exploration (35). The second model is to conduct animal experiments after fecal microbiota transplantation (FMT) based on describing the differences in intestinal microbiota composition between the disease group and the healthy group, to explore the influence of intestinal microbiota on the body. The study of Li et al. can serve as a typical case (36). First, they described differences in the composition of the gut microbiota between patients with unruptured intracranial aneurysms (UIA) and healthy people, then fed a mixture of fecal bacteria from both groups to mice that had undergone aneurysmal induction surgery, and finally found a significant increase in aneurysm rupture in mice fed stool from patients with UIA. Furthermore, they finally isolated *Hungatella hathewayi* and introduced taurine as an intermediate substance, suggesting that supplementation with *Hungatella hathewayi* and taurine could prevent the development of UIAs (36). Such studies of isolating individual strains have gradually become mainstream and are a key step in further exploring the impact of gut microbiota on disease. In addition, the researchers also fed the experimental animals a mix of antibiotics to achieve the consumption of intestinal microbiota and compared the experimental result of intestinal flora consumption and non-consumption, to illustrate the impact of intestinal microbiota on disease or physiological status (37). These three models cover most of the reported studies and are important ways to explore the relationship between gut microbiota and the human body.

As more and more research has been devoted to the gut microbiome, its interaction with the brain has come into focus in neuroscience (38). Robust evidence shows that gut microbes are linked to the central nervous system through at least three mechanisms: neural, endocrine, and immune signaling (39). Recently, Needham et al. reported that the gut microbial metabolite 4-ethylphenol (4EP) can enter the mouse brain after sulfation to 4-ethylphenol (4EPS), disrupting oligodendrocyte and myelination pattern maturation in the brain, and increasing anxiety-like behavior in mice (40). Javier et al. took a different approach, they found that *Lactobacillus rhamnosus* (JB-1) increased the expression of gamma-aminobutyric acid (GABA) receptors in the cerebral cortex of mice and had beneficial effects in the treatment of depression and anxiety, while subphrenic vagotomy prevented the antianxiety and antidepressant effects of *Lactobacillus* and GABA changes (41). In studies involving brain tumor diseases, Giuseppina et al. found that changes in gut microbiota caused by antibiotic treatment could affect the growth of glioma in mice, induce early damage to natural killer(NK)cells, and induce changes in microglial phenotype (42). On the other hand, changes in the nervous system can also cause changes in the gut microbiota (34, 43, 44). In conclusion, the most prominent communication pathways in the gut-brain axis are the vagus nerve, tryptophan metabolites, and microbial metabolites (14, 45). The communication network between the gut and the central nervous system is complex and needs to be explored.

## 4 Possible mechanisms linking gut microbiota to PitNETs

### 4.1 Endocrine system

The HPA axis is the most interesting part of the gut-brain axis when we want to explore the relationship between gut microbiota (46). Proper functioning of the HPA axis is essential for maintaining mental and physical health (47). When the function of the HPA axis is abnormal, it will affect the health status of the human body, and a large number of previous studies have proved that it is closely related to depression, bipolar disorder, depression, and other mental diseases (47, 48). In the initial stage of life, microorganisms have the function of regulating the stress response of the HPA axis (49). Social bias in germ-free (GF) and antibiotic-treated (ABX) mice have been reported to be associated with increased levels of the stress hormone corticosterone, which is produced primarily by activation of the HPA axis, and these changes are primarily through changes in neural activity rather than neural circuitry, whereas *Enterococcus faecalis* promotes social activity and reduces corticosterone levels in mice after social stress (16). Patients with Cushing’s disease and acromegalyhave a varying prevalence of psychiatric disorders, according to a systematic literature review of neuropsychological conditions in patients with PitNETs (50, 51). In addition, prolactinoma patients had a higher frequency of depressive symptoms, anxiety, and feelings of hostility (52, 53). This is due to the pituitary tumor itself, treatment, and/or long-term effects of hormonal changes on the hypothalamic-pituitary terminal organ axis (54). Especially in Cushing’s disease, the onset of psychiatric symptoms may be caused by changes in cortisol levels in the body (55). Excessive cortisol secretion, as an indicator of abnormal HPA axis function, can aggravate the symptoms of mental disorders (56). Numerous preclinical studies have demonstrated that colonization of certain types of bacteria can improve psychiatric symptoms (57–59). For example, *Bifidobacterium infantile* has been shown to improve behavioral deficits and restore basal norepinephrine (NA) concentrations (60). Messaoudi et al. showed that treatment with *Lactobacillus helveticus R0052* and *Bifidobacterium longum R0175* improved mental status and reduced cortisol levels (61). Therefore, whether gut microbes can improve the mental status of patients with PitNETs and improve hormone secretion in patients with functional PitNETs is an interesting entry point, and the HPA axis may be the key. At the same time, whether abnormal hormone secretion of functional PitNETs and gut microbiota can be linked through the HPA axis is the next research focus.

Somatotropinoma is characterized by excessive secretion of GH and increased circulatingIGF-1 concentration (62). The studies mentioned above have confirmed that the gut microbiota composition of patients with somatotropinoma is specific and correlated with GH/IGF-1 levels (7, 9). In animal studies, the researchers found whether GH or IGF-1 levels were reduced in GF mice (63–65). In turn, supplementation of GF mice or suckling piglets with gut microbiota caused corresponding increases in GH and IGF-1 levels (66, 67). Either the change of GH/IGF-1 and intestinal microbiota will lead to the change on the other side (68). It is reasonable to believe that the association between somatotropinoma and intestinal microbiota is related to the change in GH/IGF-1 level.

Intestinal microbiota performs various physiological functions in thyroxine metabolism (69, 70). Gut microbiota can directly affect thyroid hormone levels through its deiodinase activity and thyroid-stimulating hormone inhibition (71). It can also indirectly affect thyroid hormone synthesis by affecting iodine absorption (71). Therefore, it is worth exploring whether there is a relationship between the inappropriate secretion of thyroid-stimulating hormone in thyrotropinoma patients and intestinal flora.

The use of Ames mice provides an animal model lacking several pituitary hormones, including GH, thyroid-stimulating hormone, and prolactin(PRL) (72). The study by Denise et al. showed a change in gut microbiota composition in Ames mice compared to controls (72). Patients with PitNETs are likely to have endocrine dysfunction before or after surgery, manifested by the disturbance of one or more hormones (1, 73). What is the effect of this on the gut microbiota of these patients?

### 4.2 Immune system

The presence of gut microbiota can promote the development of the innate immune system (74). For instance, the gut microbiota is involved in the maturation of innate lymphocytes (ILCs) (75). Furthermore, a large number of literature has shown the presence of intestinal flora in both humoral and cellular immunity (76, 77). Takeshi et al. reported that 11 microbiota, including *Parabacteroides distasonis* and *Parabacteroides Johnsonii*, could co-induce CD8^+^T cell expression in mice and effectively inhibit tumor growth (78). Intestinal microbiota can promote the induction of immunoglobulin A (IgA) B cells and plasma cell differentiation (79). Reliable studies have also demonstrated that gut microbiota can affect the levels of immune effectors such as interleukin (IL), interferon (IFN), and tumor necrosis factor (TNF), and participate in the process of disease occurrence or development (80–84). In PitNETs, the distribution of immune cells varies among subtypes. Studies showed that macrophages, T lymphocytes, and other immune cells were more infiltrated in functional PitNETs than in NF-PitNETs (85, 86). In a study of pituitary tumors, researchers found that IFN-α significantly inhibited the secretion of functional pituitary adenoma hormones (87). TNF-α in invasive pituitary adenoma can promote pathological osteoclast formation by directly inducing osteoclast differentiation, leading to inflammatory bone destruction (88). Qiu et al. reported that serum levels of IL-4, IL-5, and IL-17 were significantly increased in patients with pituitary adenoma, and IL-17 may be an important marker related to tumor invasiveness (89). In our previous study, it was demonstrated that the gut microbiota of patients with somatotropinoma can affect the immune indexes of tumor mouse models, which is that intestinal microbes from patients with somatotropinoma promoted the growth of subcutaneous tumors in mice and up-regulated the number of programmed cell death-ligand 1 (PD-L1) positive cells in tumor tissue (8). In other types of brain tumors, gut microbiota can also affect tumors through the immune system (42). In addition, in a hot area of research called immune checkpoints, Kemeny et al. used anti-PD-L1 treatment to successfully reduce plasma adrenoceptor ticotropic hormore (ACTH) levels, delay tumor growth, and improve mouse survival in a model of Cushing’s disease (19). A large body of research evidence also suggests that there is a close relationship between gut microbiota and the response to immune checkpoint therapy. For example, bifidobacterium can enhance the therapeutic effect ofPD-L1 inhibitors on mouse melanoma, while enhancing dendritic cell function and CD8^+^T cell-mediated anti-tumor mechanisms (90).

### 4.3 Metabolism and metabolites

The gut microbiota is capable of producing and releasing active metabolites that act as signaling molecules in the brain-gut axis (91). Bacterial metabolites such as dopamine, serotonin/norepinephrine GABA, acetylcholine, and histamine can also act as neurotransmitters in the central nervous system (91, 92). Specifically, dopamine acts as both a neurotransmitter and a hormone in the hypothalamic-pituitary axis (93). First, chronic dopamine deficiency is associated with the formation of pituitary tumors and many sites in the dopamine-D2 –second receptor-second messenger pathway may be involved (94, 95). Second, dopamine binds to dopamine receptor type 2 (D2DR) in the anterior pituitary gland, thereby inhibiting hormone secretion and cell division in the anterior pituitary gland (93). Finally, dopamine agonists (DAs), by binding to D2DR, lead to the inhibition of hormone secretion and tumor shrinkage in different pituitary tumor tissue types (96). As another important substance, short-chain fatty acids (SCFAs) are produced by the gut microbiota during the fermentation of partial and non-digested polysaccharides (97). Wang et al. showed that SCFAs decreased cyclic adenosine monophosphate (cAMP) levels and subsequently protein kinase A(PKA) activity in the anterior pituitary cells of dairy cows. Inhibition of PKA activity decreased cyclic-AMP response binding protein (CREB) phosphorylation, which inhibited GH and PRL gene transcription (98). Furthermore, patients with PitNETs often have metabolic disorders associated with impaired glucose tolerance including insulin sensitivity (99–101). A variety of gut microbes regulate insulin sensitivity, like *Firmicutes*, which can produce butyrate and increases insulin sensitivity (102, 103). At the same time, microbial metabolites are usually associated with immunity, hormone levels, and so on, which is a complex mechanism of joint action (104–107).

## 5 Gut microbes and other neuroendocrine tumors

Neuroendocrine tumor (NET) is a heterogeneous group of tumors originating from different neuroendocrine organs or cells (108). In addition to PitNETs, the NETs in other organs have also been linked to gut microbes (109). By studying stool samples from 18 patients with rectal neuroendocrine tumor (RNET) and 40 controls, Hu et al. found that patients with RNET had aberrant depletion and attenuated connection. Finally, they suggest that this disordered ecological structure may contribute to the disease-causing process of this tumor (110). In gastroenteropancreatic neuroendocrine tumors (GEP-NETs), the researchers found a significant decrease in bacterial species and an increase in fungi in the patients’ gut microbes. These changes may participate in the disease process by influencing the tumor microenvironment (111). Similar to the study of PitNETs, although little has been reported so far, the relationship between gut microbes and NETs has gradually become a focus of researchers and the tumor microenvironment may be a key factor.

## 6 Conclusion

**Figure 2:**
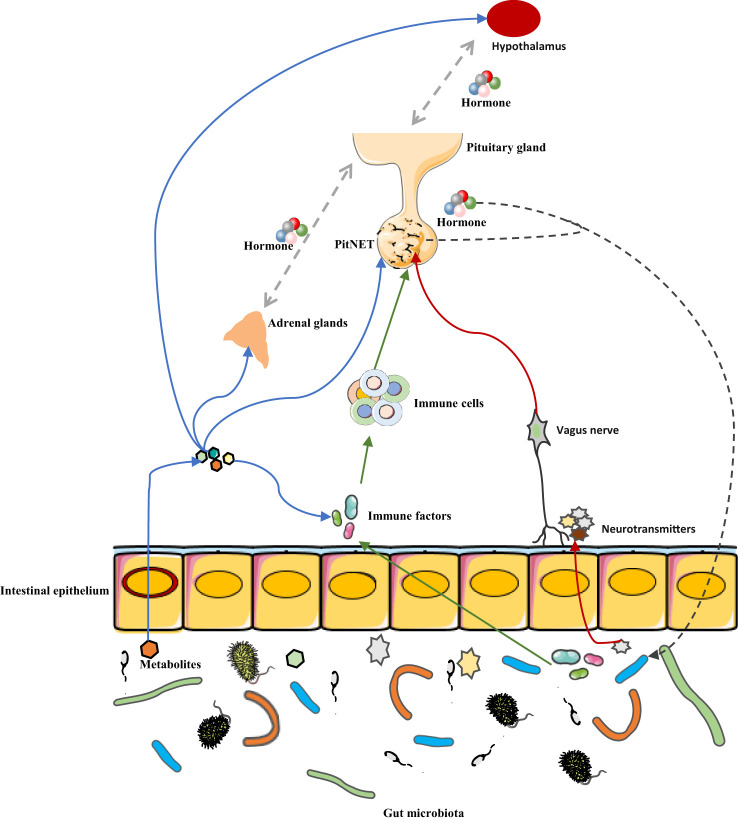
Potential mechanism of association between gut microbiota and PitNET. The interaction between gut microbes and PitNET may be mediated by immune factors, metabolites, hormone secretion, and neurotransmitters. Blue arrow, metabolites of gut microbes can not only affect PitNET by affecting immune factors, HPA axis hormone secretion, but also directly affect PitNET. Green arrow, gut microbes affect immune factors, which in turn affect immune cells in the tumor microenvironment, and ultimately PitNET. Red arrow, gut microbes release neurotransmitters that affect PitNET through the vagus pathway. Black arrow, PitNET, and the hormones it secretes can affect the composition or metabolism of gut microbes.

Patients with different types of PitNETs have their characteristics of intestinal microbial composition and can be distinguished by this characteristic. As a specific type of brain tumor, PitNETs are closely related to hormone secretion, metabolism, and the immune system. These factors are the mediators of the connection between the gut microbiota and the central nervous system, so, the link between PitNETs and gut microbes may be mediated by metabolites, hormones, and immune molecules. Thus, the future exploration of the relationship between the two parties is promising, such as whether intestinal microbes are involved in the occurrence and development of PitNETs, whether intestinal microbes can affect the clinical symptoms of PitNETs, and the influence of intestinal microbes on the immunotherapy of PitNETs, but it also means more work and challenges (**Figure 2**). According to current research, immunity and metabolism are perhaps the most important areas of concern. Expanding the number of cases and further studying the effect of intestinal microbes on tumors and its mechanism based on animal models may be the direction of further research.

## Author contributions

DN: Writing – original draft. CL: Resources, Writing – review & editing. YZ: Project administration, Writing – review & editing.
